# Right-sizing interprofessional team training for serious-illness communication: A strength-based approach

**DOI:** 10.1016/j.pecinn.2024.100267

**Published:** 2024-02-16

**Authors:** Liana Eskola, Ethan Silverman, Sarah Rogers, Amy Zelenski

**Affiliations:** a Department of Medicine, Department of Veterans Affairs, William S. Middleton Memorial Hospital and Clinics, 2500 Overlook Terrace, Madison, WI 53705, USA; b Department of Medicine, Division of Hematology, Medical Oncology, and Palliative Care, University of Wisconsin School of Medicine and Public Health, 600 Highland Ave., Madison, WI 53792, USA; c Department of Medicine, Section of Palliative Care and Medical Ethics, University of Pittsburgh, 200 Lothrop St., Suite 933W, Pittsburgh, PA 15213, USA; d Department of Social Work, Department of Veterans Affairs, William S. Middleton Memorial Hospital and Clinics, 2500 Overlook Terrace, Madison, WI 53705, United States of America; e Department of Mental Health, Department of Veterans Affairs, William S. Middleton Memorial Hospital and Clinics, 2500 Overlook Terrace, Madison, WI 53705, United States of America; f Department of Medicine, University of Wisconsin School of Medicine and Public Health, 600 Highland Ave., Madison, WI 53792, USA

**Keywords:** Communication, Training, Interprofessional, Team, Simulation, Palliative

## Abstract

**Objective:**

Palliative care communication skills help tailor care to patients' goals. With a palliative care physician shortage, non-physicians must gain these serious illness communication skills. Historically, trainings have targeted physician-only groups; our goal was to train interprofessional teams.

**Methods:**

Workshops were conducted to teach palliative care communication skills and interprofessional communication. Participants completed surveys which included questions from the Interpersonal Reactivity Index, the Ekman Faces tool, the Consultation and Relational Empathy measure, open-ended questions about empathy, and measures of effective interprofessional practice.

**Results:**

Participants felt the workshop improved their ability to listen (*p* < 0.001), understand patients' concerns (p < 0.001), and show compassion (*p* = 0.008). It increased the perceived value of peer observation (p < 0.001) and ability to reflect (*p* = 0.02) during complex conversations. Different types of professionals adopted different communication goals, though all affirmed the importance of active listening. Participants felt they improved their ability to work within an interprofessional team.

**Conclusions:**

The course effectively trained 71 clinicians, the majority non-physicians, in serious illness communication and interprofessional team communication skills, and could be reproduced in similar settings.

**Innovation:**

We adapted an approach common to physician-only trainings to diverse interprofessional groups, added a team-based component using Applied Improvisation, and demonstrated its effectiveness.

## Highlights

• Serious-illness communication skills are high-value yet resource-intensive to teach.
• Physician-only training models fall short of meeting the real-world need.
• Interprofessional teams can be trained quickly with our model.
• Participants had areas of learning overlap and divergence that met their needs.
• Majority of participants found the interprofessional training setting valuable.

## 1 Introduction

Palliative care improves quality of life for patients who have serious illnesses. One important component is palliative care specialist-led goals of care conversations, via which clinicians facilitate the delivery of care that aligns with patients' values (i.e., goal-concordant care). As the US population ages, we increasingly need a healthcare system that provides goal-concordant care. Yet the current rate at which palliative care physicians are being trained is insufficient to meet demand [1]. Thus, it is important that primary palliative care – palliative care interventions provided by non-palliative care practitioners – be implemented by all clinicians who interact with seriously ill patients [[2], [3], [4], [5], [6]], in the form of serious illness communication.

Orienting treatments to patients' goals using serious illness communication includes compassionately listening to patients in their times of grief [7] and “eliciting, articulating, supporting, understanding, communicating, interpreting, specifying, and documenting patients' [goals of care]” [8]. Despite many physicians documenting about “goals of care,” the conversations described in patients' charts instead tend to reflect situations in which doctors suspect a patient has a poor prognosis or place limitations on care, rather than documenting patient-centric goals. The real-world use of the phrase “goals of care” may not involve the application of patients' values [8]. Because applying patients' values to their care may entail iterative discussions with clinicians from multiple disciplines [9], clinicians from a variety of professions must obtain the palliative care communication skills necessary to foster goals of care discussions in serious illness communication situations.

Simulation-based medical education is an effective method for teaching complex skills to healthcare workers [10,11], and allow participants to practice a defined skill in a controlled environment that closely replicates clinical complexity. While simulations involving participants from multiple professions are common in some settings [12], few focus on solely on communication. The most common mode of simulation in health professions education uses a standardized patient (SP), who is often a trained actor. A variety of physician-only communication simulations have used a format that involves pausing mid-scenario, with a coach highlighting certain skills and areas of opportunity, and then having the participant re-enter the scenario to try new, improved skills [[13], [14], [15]]. This rapidly builds the participant's skill and creates durable learning [16]. However, only a few simulation-based palliative care communication workshops have included various members of interprofessional teams [[17], [18], [19]].

Interprofessional teams demand clinicians navigate hierarchy while working toward a common goal. The interdisciplinary nature of palliative care teams can be a model for working across difference, yet how best to confront professional culture and role expectations remains a challenge [20]. The concept of compassionate collaborative care may prove to be the “secret sauce” of high-functioning palliative care teams that can be operationalized by other teams [20]. It is difficult to measure, and procedures for integrating it are unknown, yet palliative care teams are considered to fundamentally apply it [21].

Applied improvisation is an emerging approach to improving communication and empathy amongst healthcare workers [22,23] using principles and exercises from improvisational theater to teach skills demonstrated by high-functioning teams. The three main skills of medical improv are attunement, affirmation, and advancement [24]. Building on the “yes, and” concept from Viola Spolin, one must first attune to themselves and the environment, then affirm what is already there, and lastly use this information to advance the situation for all involved. The “micro” skill under each of these overarching skills is active listening which encompasses a desire to listen for what is underneath the words one is hearing. What is the meaning behind what the other person is sharing? Improv is well suited for practicing these skills because practitioners are working without a script as they build a reality together out of thin air. They must work as a team by listening to co-create the meaning and content for their scene. This is also an essential skill for goals of care conversations where a clinician is attempting to uncover what is truly important to a patient in a difficult new health situation in order to plot the course forward.

Any potential synergy of serious illness communication training using palliative care techniques and Applied Improv to produce clinicians more disposed to compassionate collaborative care has not been studied previously to the best of our knowledge. Nor has the use of Applied Improv been studied in post-training (in-practice) interdisciplinary groups, upon our review of the literature.

We created a serious illness communication simulation workshop for non-palliative care clinicians from multiple professions using customizable cases with real-world fidelity. This approach was necessary for our small veterans' health care environment (129 beds, 2.1 FTE palliative care physicians), and is applicable to similar settings which lack many palliative care clinicians yet have a great need for the skill sets embodied by palliative care specialists. We also included Applied Improvisation exercises to improve participants' interprofessional communication skills, which are considered an essential competency as determined by the Institute of Medicine [25] (now the National Academy of Medicine).

The application of core palliative care communication skills like asking about perception of illness, providing clinical empathy, and inquiring about patients' and families' goals and priorities, can foster longitudinal goals of care discussions. We posited that our approach to conveying these skills to a multi-disciplinary audience, utilizing live simulation exercises and Applied Improvisation exercises combined into single-day workshops, would be a novel, effective way to facilitate skills development.

## 2 Methods

### 2.1 Setting and participants

The Veterans Health Administration (VA) is the largest integrated health care system in the United States. VA commits resources and expertise to palliative and end-of-life care through its funding of dedicated staff, educational initiatives,s and research [26]. Clinicians working in inpatient hospice, inpatient acute care, outpatient primary care, and specialty clinic settings at the William S. Middleton Memorial Veterans Hospital and Clinics in Madison, Wisconsin were invited to participate in the workshops. Participants were recruited broadly in order to maximize the “reach” of this intervention, rather than training individuals in only certain specialty settings or professions. Information about specific practice settings was not collected.

Coaches were recruited from the University of Wisconsin Section of Palliative Medicine, Meriter Hospital, and Agrace, a local not-for-profit hospice agency in southern Wisconsin. Amongst the eight coaches were seven fellowship-trained palliative medicine physicians and one medical educator with extensive experience facilitating similar workshops (AZ).

### 2.2 Workshop description

A total of four workshops were scheduled in October and November 2019 according to coaches' availability. Content was chosen based on a mandate from the Veteran's Health Administration to apply grant funding to improve the quality of end-of-life care for veterans, published literature, one author's prior experience with effective workshop teaching of this kind, and discussions with stakeholder leadership. Each workshop included 6.5 contact hours of instruction in non-clinical meeting spaces on the University of Wisconsin campus. From amongst the group of eight coaches, different sets of four taught during each workshop. Only two coaches (authors LE and ES) taught in all four workshops. The training was priced at $450 per participant and fees were paid on their behalf by the Madison VA Hospital to the University of Wisconsin. In addition to eight coaches and six actors, one administrative professional was involved to coordinate the workshops, payments, actor hiring, room reservations, two meals per workshop for those present, course surveys, and workshop materials (including printed cognitive aids for participants and printed cases for actors and coaches).

One coach (author LE) began each workshop session with an 80-min lecture (available upon request) to introduce participants to the principles of palliative care, simulation-based communication education, adult learning theory, and topics specific to communicating about serious illness. Specific cognitive maps described in the didactic session included SPIKES [27] for breaking bad news, NURSE [28] for responding empathically to strong emotions, and REMAP [29] for conducting goals of care conversations in part or in full. Each of these tools is used extensively in palliative care education across the United States. Participants were given printed handouts describing each tool and how to use it. Each session's group of between 17 and 20 people was then divided into four smaller sets of four or five participants, each led by a coach. All coaches but one had specialized experience in this type of small group simulation facilitation, either via formalized training from the VitalTalk organization, or previous experience teaching in similar workshop settings. One coach (ES), a palliative care medical education fellow, led their small groups under the guidance of a senior palliative care physician.

Two authors (LE, ES) adapted cases originally developed for a regional “Delivering Difficult News course” called “PalliTALK” (https://www.medicine.wisc.edu/hematology-oncology/pallitalk-and-wetalk-communication-workshops) to include pertinent details for interprofessional colleagues. Cases were originally written by a medical educator (AZ) and a palliative medicine physician circa 2011 based on real-life patient situations (altered extensively to preserve confidentiality). Each of five cases focused on a different character and disease process, and the main character from each case was either the patient or other medical decision-maker. Most cases originally presumed the clinician was a palliative care physician in a hospital, but were revised to presume the clinician could be from a variety of professions and in various settings according to a participant's usual work setting and role. Cases included information on the clinical situation, characters, and the task at hand (usually discussing a change in condition). Cases are available upon request.

Professionally trained actors played the patient or decision-making character. Their previous training included an unrecorded number of hours of experience playing their character as described in each case in previous PalliTALK workshops. Actors had a 90-min refresher session in October 2019 prior to the first workshop. During each workshop, actors rotated through the rooms where each small group was based, with one “green room” in the rotation where each actor had a timed break. A total of six actors played roles in the workshop series, but only five per session.

During the simulations, each participant took a turn in the “hot seat” interacting with a character while the other small group members and coach observed, taking notes to provide behavioral feedback. The participant leading the conversation or the coach could call a “time-out” at any time during the simulation. The coach would ask for the participant's thoughts on the interaction, then turn to the small group for specific observations before adding their own feedback. Working with the coach and small group, the participant would identify a specific skill they wished to apply, and then the scenario would either “rewind” to where they stopped, or skip ahead, so they could try the identified approach. The goal was for each participant to work at the leading edge of their skill level by having the coach and actor challenge them appropriately [30]. At the conclusion of the interaction, the coach helped with learning consolidation by asking the participant to choose one skill from the simulation they would use in the future, continuing the cycle of learning as they reentered practice. This cycle repeated for each member of the small group. Participants were asked not to discuss the contents of the session to anyone not present, to promote psychological safety. After the simulations, an author trained in Applied Improvisation (AZ) led the entire group of 17–20 participants through three Applied Improv exercises, followed by debriefing sessions to explore the concepts of communication and collaboration within the healthcare team. These interactive exercises provided fresh, engaging approaches to active listening and identifying values [31].

### 2.3 Conceptual framework

Kolb's cycle of experiential learning was used to guide the teaching and evaluation methods. This process includes four iterative steps: 1) a concrete experience, 2) reflective observation, 3) abstract conceptualization, and 4) active experimentation [32]. The medical improv activities provided concrete experiences related to communication in the healthcare environment. Through the debriefs, participants reflected on their own experiences in of the activity and explored how their new insights might enable them to interact in the environment in new ways. During the small group sessions, the coach started by setting goals and having participants reflect on prior concrete experiences. Going forward, the simulated cases provided the experiences on which the participants would reflect (in dialogue with their coach and peers), then they would choose a way forward and go back into the scene to experiment with that new understanding.

### 2.4 Measurements

A systematic program evaluation of this workshop was conducted, informed by a pre-post convergent mixed methods approach. The quantitative and qualitative data were merged during the analysis phase to fully understand the impact of the workshop on the participants [33]. The goal of program evaluation is to examine a program or learning activity to measure change in the participants' knowledge, skills, attitudes, and/or behaviors [34]. A research study was not conducted; rather, research methods to examine the outcomes of the workshop were used.

The Interpersonal Reactivity Index and an assessment based on the Ekman Faces tool were used to measure dispositional empathy and emotion recognition respectively at baseline [35,36]. The IRI is a 28-item survey used to assess four domains of empathy: 1) Perspective-taking, 2) Empathic Concern, 3) Fantasy, and 4) Personal Distress [35]. The IRI is a valid and reliable tool to measure empathy, however it has been shown not to be responsive to intervention in the short term. Therefore, we used it only as a premeasure to assess baseline differences amongst the participants. Similarly, participants completed a non-validated 19-item multiple choice survey asking them to identify emotions through facial expressions based on the Ekman Facial Action Coding System to assess their accuracy of naming emotions [37]. Participants also rated their ability to change a communication plan in real-time if things are not going well on the 5-point scale. This question was intended to measure their ability to reflect in action, which could be a powerful tool and enhance the reflective observation in the Kolb cycle [32]. The pre-survey also contained two open-ended questions about the utility of empathy in the clinical encounter and methods used to express empathy. Lasty, participants answered simple demographic questions including gender, training level, and degree.

To measure change, participants completed a pre survey immediately before the workshop and a post survey at the conclusion of the workshop. This survey included a modified version of the Consultative and Relational Empathy (CARE) measure. Participants rated their skill on a 5-point scale (1 = Poor, 2 = Fair, 3 = Good, 4 = Very Good, 5 = Excellent) in five areas: 1) explaining things clearly, 2) letting [the patients] tell their whole story, 3) showing care and compassion, 4) making a plan of action, and 5) fully understanding their concerns [38]. Though the CARE measure was originally validated as a patient-reported measure, it has also been validated as self-report tool as used here [39].

In addition to the CARE measure, the post survey included questions about the educational quality of the workshop and how the activities contributed to effectiveness working in an interprofessional team, the latter via four domains: “Applying values/ethics to interprofessional practice,” “Defining the roles/responsibilities of my team members,” “Engaging in effective interprofessional communication,” and “Working with an interprofessional team.” Finally, participants identified one or two behaviors they would like to change or improve in the future based on the workshop.

The IRI and CARE measures are both validated tools. The other questions were developed by the evaluation team to assess other skills related to the workshop content for which the team was unaware of validated measures and to examine the participants' reactions to the workshop.

Emotion recognition and empathy measures were chosen due to the importance of empathy in goals of care conversations and the lack of ability to measure these conversations inside or outside of the workshop. The workshops were non-evaluative for the participants so that they felt more comfortable trying new skills and engaging in active experimentation. Empathy skills are key for having conversations centered on patient goals and priorities. Empathic opportunities abound in serious illness conversations, so it is an important skill focus. The CARE measure in particular lends itself to goals of care conversations by focusing on listening and showing care and concern [40].

### 2.5 Data collection and analysis

An administrative coordinator administered the pre- and postprogram assessments during the workshop using an online survey (available upon request). Participants accessed the survey through a QR code sent to their email accounts on their mobile devices

All continuous variables were summarized as mean plus standard deviation. A three-way analysis was conducted on Table 2 using the analysis of variance (ANOVA) for normally distributed variables and the non-parametric Kruskal Wallis test for non-normal data. Repeated differences were examined using the Wilcoxon sign rank test in Table 4 since the data were not normally distributed. Data normality was tested for using histograms and the Bartlett test. Paired effect sizes were conducted based on mean comparisons using the Cohen's d test (Effect size Scale: ignored (d < 0.2), small (d = 0.2), moderate (d = 0.5), and large (d ≥ 0.8). All *p*-values were considered statistically significant at *p* ≤ 0.05. All analyses were conducted using STATA version 17 (StataCorp. 2021. Stata Statistical Software: Release 17. College Station, TX: StataCorp LLC).

Responses to open-ended questions were subjected to thematic content analysis [41], which is a qualitative technique to identify emergent themes. This technique was chosen to assess what participants felt the course “did” for them and has been applied in other settings where Applied Improvisation exercises have been used [22]. Using Nvivo qualitative software (QSR International Pty Ltd. (2020) NVivo (released in March 2020), https://www.qsrinternational.com/nvivo-qualitative-data-analysis-software/home), all four authors engaged in consensus coding to characterize the data from participants. Once the data were coded, they were organized by professional group (“Providers,” Nurses, and Social Workers) to uncover any differences based on role. The “provider” category included people possessing the qualifications of MD, APNP, DNP, “NP,” APRN, or PA. Members of the “nurse” category included people with the degrees of BSN, RN, or MSN. The “social worker” group included people with the qualifications of LCSW, MSW, or “social work.”

This work was deemed exempt by the University of Wisconsin-Madison IRB.

## 3 Results

### 3.1 Participants

A total of seventy-one clinicians participated in four workshops in October and November 2019, with each participant attending a single workshop session. Eighty-nine percent (*n* = 66) identified as female and 7% (*n* = 5) identified as male (Table 1). Participants reported a wide variety of professional degrees and qualifications (Table 1) which were condensed into three categories: Nurse (35%, *n* = 26), Provider (22%, *n* = 16), and Social Worker (36%, *n* = 27). Two of the 71 participants did not fit into a category, therefore, they were excluded from professions comparisons. Their data were included in all other analyses.

**Table 1:** Demographic characteristics of workshop participants.

	Attended *n* = 71	Completed Pre-Survey *N* = 58	Completed Post-Survey *N* = 62
Characteristics	Number (%)	Number (%)	Number (%)
Gender			
Female	66 (93)	55 (94.8)	57 (91.9)
Male	5 (7)	3 (5.2)	5 (8.1)
Degree			
APNP	1 (1.4)	0	1 (1.6)
APRN	1 (1.4)	1 (1.7)	1 (1.6)
BA	1 (1.4)	1 (1.7)	1 (1.6)
BSN, RN	7 (9.9)	7 (12.1)	7 (11.3)
DNP	4 (5.6)	4 (6.9)	3 (4.8)
LCSW	2 (2.8)	2 (3.4)	2 (3.2)
MD	2 (2.8)	1 (1.7)	2 (3.2)
MOT	1 (1.4)	1 (1.7)	1 (1.6)
MSN, RN	2 (2.8)	1 (1.7)	1 (1.6)
MSW	24 (33.8)	21 (36.2)	22 (35.5)
NP	4 (5.6)	3 (5.2)	4 (6.5)
PA	3 (4.2)	3 (5.2)	3 (4.8)
PharmD	1 (1.4)	1 (1.7)	1 (1.6)
PhD	1 (1.4)	1 (1.7)	1 (1.6)
RN	16 (22.5)	11 (19.0)	10 (16.1)
Did not reply	1 (1.4)	1 (1.7)	1 (1.6)

### 3.2 Quantitative measures

All *p*-values were considered statistically significant at *p* ≤ 0.05. No significant differences were found in any category at baseline for the IRI, CARE, and Ekman instruments (Table 2). Significant changes were seen from pre to post in the following domains of the CARE measure: “Listen to the patient's whole story” (*p* < 0.001), “Understand [patients'] concerns” (p < 0.001), and “Show care and compassion” (*p* = 0.008) (Table 3). A significant increase was detected in the perceived value of peer observation (p < 0.001) and ability to reflect in action (*p* = 0.02) after the training (Table 3).

**Table 2:** Baseline comparison of IRI and Ekman Survey by Profession.⁎

	Nursing N = 26	Provider N = 16	Social Work N = 27	P
Pre EC, mean (SD)	21.6 (3.7)	23.4 (3.4)	22.3 (3.7)	0.35a
Pre PD, mean (SD)	9.2 (3.9)	11.7 (5.5)	9.4 (4.3)	0.20a
Pre PT, mean (SD)	19 (3.9)	19.1 (3.3)	20.8 (4.2)	0.24a
Pre FS, mean (SD)	18.6 (5.8)	15.9 (4.4)	16 (5.7)	0.21a
Ekman Score, mean (SD)	13.5 (1.5)	12.1 (2.1)	12.9 (2.4)	0.13a

A = analysis of variance.

B=Kruskal Wallis.

⁎ statistically significant at p ≤ 0.05.

**Table 3:** Pre/Post comparison of CARE measure items.

	Total, N = 71				
	Pre	Post	P	ES	Interpretation
Explain	3.1 (0.67)	3.4 (1.10)	0.13	0.34	small
Plan	3.1 (0.93)	3.1 (1.30)	0.84	0.03	ignored
Reflection in Action	2.9 (0.92)	3.3 (1.10)	**0.02**⁎	**0.50**	moderate
Story	3.3 (0.89)	3.8 (0.93)	**<0.001**⁎	**0.64**	moderate
Understand	3.0 (0.79)	3.7 (0.98)	**<0.001**⁎	**0.76**	moderate
Care and Compassion	3.7 (0.81)	4.1 (0.92)	**0.008**⁎	**0.43**	small

Effect size Scale: ignored (d < 0.2), small (d = 0.2), moderate (d = 0.5), and large (d ≥ 0.8).

⁎ Statistically significant at *p* ≤ 0.05.

**Table 4:** Pre/post comparison by professional group.

	Nursing, N = 26			Provider, *N* = 16			Social Work, *N* = 27		
	Pre	Post	P	Pre	Post	P	Pre	Post	P
Explain	2.8 (0.69)	3.4 (0.83)	0.11	3 (0.60)	3.6 (0.5)	**0.02**⁎	3.5 (0.51)	3.4 (1.4)	0.66
Plan	2.7 (1.0)	2.7 (1.2)	1.0	3 (0.85)	3.2 (1.3)	0.52	3.5 (0.72)	3.5 (1.3)	0.78
Reflection in Action	2.3 (0.78)	3.7 (0.95)	0.11	3.3 (0.70)	3.4 (1.3)	**0.002**⁎	3 (1.4)	2.8 (1.1)	0.34
Story	2.9 (0.99)	3.8 (0.54)	0.09	3.7 (0.75)	4.0 (1.0)	**0.008**⁎	3 (1.4)	3.8 (1.1)	**0.05**⁎
Understand	2.7 (0.78)	3.8 (0.83)	**0.004**⁎	3.5 (0.59)	3.9 (1.1)	**0.004**⁎	2.5 (0.71)	3.4 (1.1)	**0.04**⁎
Care and Compassion	3.7 (0.65)	4.1 (0.62)	0.28	3.8 (0.78)	4.2 (1.0)	**0.03**⁎	3 (1.4)	3.8 (1.1)	**0.11**

Wilcoxon sign rank test.

⁎ Statistically significant at p ≤ 0.05.

**Table 5:** Representative reponses to open-ended questions.

Category	Representative Response
Listening	Be intentional about getting patient/family's perspective/understanding before jumping in with my own.
Goal Oriented Communication	Use the golden questions to elicit better understanding of where the client is at and future goals
Compassionate Presence	Ability to sit with emotions and not rush to fix things or offer solutions.
Reflection (Reflective statement)	Rephrasing what they say so they know I've heard them.
Building Relationship	Find common ground or a connection to build a conversation from.

A majority of participants agreed this workshop contributed to the four domains of professional effectiveness for them: 81.2% agreed or strongly agreed it improved their interprofessional communication; 79.7% agreed or strongly agreed it helped them work in an interprofessional team; 73.8% agreed or strongly agreed it helped them apply values and ethics to interprofessional practice; and 68.1% agreed or strongly agreed it helped define the roles and responsibilities of their team members. A consistent 13% of respondents disagreed that the workshop contributed to the professional effectiveness domains.

### 3.3 Open-ended questions

Representative responses are shown in Table 5. When asked about behaviors and skills contributing to empathy that they wanted to continue to practice after the workshop, participants mentioned listening (“Listening”) most frequently. Following that, in descending order of prevalence, were communicating about goals of care (“Goals”), maintaining a therapeutic presence (“Presence”), using reflective statements (“Reflection”), and fostering good relationships with patients (“Relationship”). Professional groups differed, however, in the frequency with which specific skills were mentioned. Social Workers mentioned “Presence” second most often and Goals third most often, whereas Providers and Nurses identified “Relationship” second most often, and “Validating” the patient and “Goals” third most often, respectively.

## 4 Discussion and conclusion

### 4.1 Discussion

We facilitated one-day workshops for professionally diverse sets of practitioners to learn empathic communication skills originating from palliative care. Most participants felt their ability to communicate compassionately in simulated serious illness situations improved. Process measures also showed improvement in participants' abilities to reflect on their behaviors and increased the perceived value of peer observation. Including Applied Improvisation exercises led to an improvement in perceived ability to work effectively within a team. In taking the quantitative and qualitative data together, it becomes clear that participants were working on their abilities to listen more intentionally, manage their presence in the room, and leverage their relationships and communication techniques to uncover the true goals of their patients moving forward. They reported the largest gains in these same skills on the CARE measure.

#### 4.1.1 Perceptions of self-efficacy in compassionate communication

Participation in our workshops significantly improved attendees' self-perceived abilities to listen to patients, understand their concerns, and show care and compassion toward them. These fundamental skills lend to the development of more complex skills, such as leading goals of care conversations, and may be considered the “secret sauce” that interdisciplinary palliative care teams use to deliver compassionate, collaborative care [21]. Because participants' abilities to reflect on their own behaviors improved, a training such as this may “prime” individuals to take those reflective behaviors back into the clinical environment. Studies have found that using structured communication tools can lead to higher rates of documentation of patients' goals and values, but experience is often a better teacher than repeated trainings [12], so the value of a single training to change attitudes (and possibly behavior) should not be underestimated.

#### 4.1.2 Improved interprofessional collaboration

Serious illness communication demands a high level of interprofessional collaboration. Workshop attendance in this case appears to have resulted in perceived improvements in several domains of interprofessional effectiveness, including communication, working within a team, applying values and ethics in teamwork, and defining roles and responsibilities. This suggests that trainings with interprofessional collaboration as a goal can create the substrate for more successful serious illness communication and compassionate collaborative care.

Because participants also experienced an increase in the perceived value of peer observation in the workshop setting, they might ask their coworkers for feedback on their communication skills in clinical settings in the future, producing more collaborative relationships and making serious illness communication “everyone's business.”

Of note, a consistent 13% of participants marked “disagree” when asked if the workshop helped with their interprofessional effectiveness, leading to the possibility that they decided the workshop was entirely unhelpful in that area (or did not read the questions).

#### 4.1.3 Implications for future empathic practices

Participants from three distinct professional groups, with differing roles in the healthcare system, were unanimous in the most important skill they would carry forward in their practice: listening. Members of all three professional groups also listed developing goals of care skills as important growth areas for them. Each group had subtle variations in which other empathic communication skills they most wanted to practice in the future. Providers and Nurses both listed relationship-building skills and validating patients in their top five. This may suggest they have not traditionally seen those areas as their strength or within their purview. Social workers, however, did not mention either relationship-building skills or validating patients in their top five, perhaps indicating these are already areas of comfort for them.

#### 4.1.4 Right-sizing serious illness communication training for interprofessional groups

We utilized tools to measure participants' empathy rather than specific tools that quantify effectiveness in goals of care conversations for a variety of reasons. First, empathy skills lead to better end-of-life care and better team-based collaboration [21]. Empathy, sharing, respect, and partnership are aspects of patient- and family-centered care that are necessary ingredients of shared decision making and goal setting, which are the “overarching processes for achieving compassionate collaborative care at end-of-life” [21]. Similar workshops [42] have produced more empathic clinicians who are more disposed to conducting goals of care conversations.

Additionally, we assessed measures of interprofessional collaboration because if we expect clinicians to work in tandem to holistically assess and implement patients' goals of care, it behooves us to determine what specific interventions facilitate that vital teamwork. Because empathic communication is a precondition for effective goals of care conversations, it is difficult to assess them separately. In real-world settings where interventions should be expected to deliver the highest-yield skills to the largest audience possible in a cost-effective manner, focusing on core skills like empathy arguably might do more to effect compassionate, collaborative care [21] than a narrower set of communication skills deployable only in certain settings, by practitioners in whom enormous resources have been concentrated (like palliative care physicians).

The convergence of learning amongst interprofessional groups suggests that one training can meet needs of a diverse range of professionals. Using medical improvisation exercises enriched the training environment and improved participants' sense of interprofessional effectiveness. In summary, this workshop could act as a guide for other institutions to improve access to palliative care-style communications skills for a diverse group of healthcare professionals and inculcate the importance of interprofessional collaboration in continuing education environments, with the goal of improving compassionate collaborative care and making serious illness communication “everyone's business.”

Of note, workshops were held prior to the COVID-19 pandemic and the prevalent shift from in-person trainings to virtual ones. There has been much success in providing similar trainings in virtual format, and we believe our workshop could be adapted thusly [43].

#### 4.1.5 Limitations

Though this work's innovations reveal new possibilities in team-based communication learning, there were limitations. While we explicitly included professional groups that often have not been targeted in palliative care communication trainings, we did not include all possible interdisciplinary team members. In addition, participant responses were self-reported, and we did not measure changes in behaviors. It is thus possible that our results resulted from positive feelings participants had about the workshop; positive interactions with instructors, other participants, and actors; or factors other than the intervention. It is also possible that our participants' changed attitudes did not translate to a change in behaviors, or improved patient outcomes, which would severely limit the applicability of this training.

Having participants evaluate themselves prior to the workshop and afterward could have resulted in response-shift bias, in which participants change the standards they use to evaluate themselves as a result of workshop participation. Literature shows that study participants “routinely overrate their knowledge or skills prior to training” and “interventions that are effective in improving knowledge or skills are likely to cause respondents to reappraise—and lower—their former assessments of their competency” [44,45]. Because a self-rating prior to a program can be an overestimate of the participant's skill level, the net effect is an underestimation of an intervention's effectiveness. However, subjecting participants to retrospective preprogram self-ratings (ratings of one's perceived behaviors prior to a training, made after a training's conclusion) rather than a conventional preprogram self-rating could have introduced a social desirability bias effect, in which the perspective gained in a training causes participants to align their perceptions of prior behaviors with their new ideal of “good” care [44]. On balance, we find the conventional pre- and post-program self-ratings to be more applicable to our situation and more likely to minimize bias.

Another potential limitation was lack of a control group. However, controlled studies have shown that communications skills did not improve in control groups [42]. More applicable is the concern that clinicians may behave differently in simulations than they do in real clinical situations [42]. Lastly, it is possible that because attendance was voluntary and we recruited broadly, participants were motivated to attend and may have had more positive attitudes about the course content than if participation had been compulsory.

### 4.2 Innovation

Palliative care communication skills are high-value, resource-intensive skills for physicians to obtain, with the gold standard being a one-year post-residency fellowship in Hospice and Palliative Medicine. Having access to such physicians to provide uniform serious illness communication services is out of reach for many smaller healthcare systems [1]. Staffing full interdisciplinary palliative care programs also remains out of reach for more than a quarter of all hospitals with >50 beds [46]. One means of addressing this is to make providing goal-concordant care “everyone's business.” Yet gaps exist in the literature regarding how to accomplish this in a simple, cost-effective manner, especially for multi-disciplinary interprofessional groups. Many studies have demonstrated the effectiveness of simulation-based palliative care communication workshops in delivering skill uptake and increasing clinician empathy [13,[15], [16], [17], [18], [19]]. Donesky et al. used a similar model to teach interprofessional collaboration skills to Palliative Medicine clinicians and effectively improved participant's attitudes about interprofessional teamwork [21], though did not include the use of medical improvisation. Our work complements Donesky's by using a team-based approach with an almost entirely non-physician audience *outside* of palliative medicine and introducing medical improvisation exercises to improve interprofessional communication and empathy [21]. Other communication trainings historically have measured the uptake of serious illness communication skills with the use of tools like SPIKES and NURSE [42] but have not included professionally diverse audiences.

### 4.3 Conclusion

This program evaluation examined the outcomes of an interprofessional group of clinicians in a palliative care communication training that incorporated team communication exercises using Applied Improvisation, and can serve as a framework for training to improve serious illness communication and interprofessional effectiveness. As noted, different types of professionals came away with differing areas of perceived improvement. Future work should focus on how to bolster communication skills unique to each professional group in an interprofesssional setting and expand competence in areas of lesser comfort. To improve goal-concordant care, more clinicians must receive training and feel comfortable with empathic conversations about patients' goals and values to make goal-concordant care “everyone's business.”

## Funding

This material is the result of work supported with resources from the Department of Veterans Affairs, Veterans Health Administration, Palliative and Hospice Care Program in the Office of Geriatrics and Extended Care (GEC). The sponsor was not involved in the study design; in the collection, analysis or interpretation of data; in the writing of this report; or in the decision to submit the article for publication.

The views expressed in this article are those of the authors and do not necessarily reflect the position or policy of the Department of Veterans Affairs or the United States government.

## CRediT authorship contribution statement

**Liana Eskola:** Writing – review & editing, Writing – original draft, Visualization, Resources, Project administration, Investigation, Conceptualization. **Ethan Silverman:** Writing – review & editing, Writing – original draft, Resources, Investigation. **Sarah Rogers:** Writing – review & editing, Writing – original draft, Project administration, Funding acquisition, Conceptualization. **Amy Zelenski:** Writing – review & editing, Writing – original draft, Visualization, Supervision, Resources, Project administration, Methodology, Investigation, Formal analysis, Data curation, Conceptualization.

## Declaration of competing interest

The authors declare that they have no known competing financial interests or personal relationships that could have appeared to influence the work reported in this paper.
